# Expression and Prognostic Significance of Human Epidermal Growth Factor Receptors 1, 2 and 3 in Periampullary Adenocarcinoma

**DOI:** 10.1371/journal.pone.0153533

**Published:** 2016-04-12

**Authors:** Jacob Elebro, Margareta Heby, Carl Fredrik Warfvinge, Björn Nodin, Jakob Eberhard, Karin Jirström

**Affiliations:** Department of Clinical Sciences, Division of Oncology and Pathology, Lund University, Skåne University Hospital, 221 85, Lund, Sweden; INRS, CANADA

## Abstract

Periampullary adenocarcinoma, including pancreatic cancer, is a heterogeneous group of tumours with dismal prognosis, for which there is an urgent need to identify novel treatment strategies. The human epithelial growth factor receptors EGFR, HER2 and HER3 have been studied in several tumour types, and HER-targeting drugs have a beneficial effect on survival in selected types of cancer. However, these effects have not been evident in pancreatic cancer, and remain unexplored in other types of periampullary cancer. The prognostic impact of HER-expression in these cancers also remains unclear. The aim of this study was therefore to examine the expression and prognostic value of EGFR, HER2 and HER3 in periampullary cancer, with particular reference to histological subtype. To this end, protein expression of EGFR, HER2 and HER3, and *HER2* gene amplification was assessed by immunohistochemistry and silver *in situ* hybridization, respectively, on tissue microarrays with tumours from 175 periampullary adenocarcinomas, with follow-up data on recurrence-free survival (RFS) and overall survival (OS) for up to 5 years. EGFR expression was similar in pancreatobiliary (PB) and intestinal (I) type tumours, but high HER2 and HER3 expression was significantly more common in I-type tumours. In PB-type cases receiving adjuvant gemcitabine, but not in untreated cases, high EGFR expression was significantly associated with a shorter OS and RFS, with a significant treatment interaction in relation to OS (p_interaction =_ 0.042). In I-type cases, high EGFR expression was associated with a shorter OS and RFS in univariable, but not in multivariable, analysis. High HER3 expression was associated with a prolonged RFS in univariable, but not in multivariable, analysis. Neither HER2 protein expression nor gene amplification was prognostic. The finding of a potential interaction between the expression of EGFR and response to adjuvant chemotherapy in PB-type tumours needs validation, and merits further study.

## Introduction

Adenocarcinomas originating in the head of the pancreas, the distal bile duct, the ampulla of Vater and the duodenum are often grouped together as periampullary tumours, since they can be difficult to distinguish from each other clinically. Pancreatic cancer is the most common type of periampullary adenocarcinoma, accounting for 3% of all cancer in the USA [1] and the Nordic countries [2] and, due to very high lethality [3], 7% of all cancer-related deaths, making it the fourth most common cause of cancer-related death in the western world [1, 2]. After surgery, periampullary adenocarcinomas have traditionally been primarily categorized according to their anatomical origin, but recent research has shown that morphological subtype is a more rational basis for classification [4]. Pancreatobiliary (PB) type morphology dominates in periampullary tumours of pancreatic and distal bile duct origin, but is also seen in the ampulla of Vater [4, 5]. They have a significantly worse prognosis than tumours with an intestinal (I) type morphology, which are mainly found in the ampulla of Vater and in the duodenum [4–6].

Members of the HER (*human epithelial growth factor receptor)* family of tyrosine kinase receptors are essential for human development and growth, and they are overexpressed in several human cancers. They consist of four closely related transmembranous molecules, EGFR (HER1, ErbB-1), HER2 (Neu, ErbB-2), HER3 (ErbB-3) and HER4 (ErbB-4). Ligand-binding causes hetero- or homodimerization of receptors and intracellular transphosphorylation, which activates several intracellular signalling-cascades important for cell survival, proliferation and growth. Combinations of HERs give dimers that vary in stability, affinity for their ligands and activation of different signalling-cascades [7].

There are several drugs, targeting either the extracellular domains or the intracellular tyrosine kinase domains of the HERs, that give survival benefits in selected cases of breast, colon, gastric and lung cancer [7], and combinations of HER-active drugs have been shown to further improve survival compared with single HER-therapy [8].

Expression of EGFR is common in pancreatic cancer, and has been associated with metastatic potential [9], but several studies have not found any prognostic effect of EGFR expression on overall survival (OS) [9–11]. A meta-analysis did however find a survival disadvantage in pancreatic cancer expressing EGFR [12]. Addition of the EGFR tyrosine kinase inhibitor erlotinib to gemcitabine lead to an increased OS in patients with advanced pancreatic cancer [13], the improvement was however modest and erlotinib is therefore rarely used for treatment of pancreatic cancer in clinical practice. Other EGFR active drugs have not led to a prolonged OS, when added to standard chemotherapy [7]. EGFR expression has also been shown to be more common in PB-type than in I-type ampullary adenocarcinoma [14] and overexpression has been associated with shorter OS in I-type but not in PB-type tumours [15].

The reported rates of HER2 overexpression in pancreatic cancer, defined as 3+ in immunohistochemical staining or gene amplification by in situ hybridization (ISH), vary from 0%-11% [16–23]. Similarly to other tumour types, high expression of HER2 in pancreatic cancer has been associated with a shorter survival [24], but other studies have found the opposite [25]. Addition of the HER2 antibody trastuzumab to gemcitabine in metastatic pancreatic cancer overexpressing HER2 (2+ or 3+) gave no clear survival benefit compared with the expected survival upon gemcitabine alone [26].

In tumours of the ampulla, distal bile duct and gall bladder, the frequency of HER2 overexpression has been low, and comparable to pancreatic cancer in a few small studies [16, 17, 23], whereas larger studies have found overexpression in 6–13% of ampullary tumours [27, 28], and 23% and 17% of tumours of the bile duct and gall bladder [29].

In ampullary adenocarcinoma, HER2 gene amplification has been equally distributed over morphological types, and amplified cases have been wild-type for KRAS, NRAS and BRAF. There has also been an equal distribution of 2+ and 3+ immunohistochemistry among ISH amplified cases [27].

In one previous study on patients with resected pancreatic cancer, high HER3 expression was denoted in 41% of cases, and was found to be associated with a shorter OS [30], which is in line with studies on several other types of adenocarcinoma [31]. However, high HER3 expression has also been found to correlate with a prolonged survival in colorectal cancer [32, 33], breast cancer [34] and in gastric and oesophageal cancer [35]. Anti-HER3 antibodies have been shown to reduce growth in pancreatic cancer cell lines that are wild-type for KRAS [36]. To the best of our knowledge, the expression and prognostic impact of HER3 has not been studied in the full spectrum of periampullary adenocarcinoma.

Thus, the prognostic role of HERs in periampullary adenocarcinoma has mostly been studied in pancreatic cancer, often with conflicting results, and less in I-type adenocarcinomas. The efficacy of treatments targeted at EGFR, HER2 and HER3 are not well studied in periampullary cancer, in particular in I-type tumours. Therefore, the aim of this study was to analyse the expression and prognostic significance of EGFR, HER2 and HER3, with particular reference to morphological subtype, in a retrospective, consecutive cohort of 175 cases with periampullary cancer.

## Materials and Methods

### Patients

The study cohort is a previously described retrospective consecutive series of pancreaticoduodenectomy specimens from all patients (n = 175) with periampullary adenocarcinoma, including pancreatic cancer, resected at the University hospitals of Lund and Malmö, Sweden, from January 1 2001 until December 31 2011 [37–41]. Data on survival were gathered from the Swedish National Civil Register. Follow-up started at the date of surgery and ended at death, at 5 years after surgery or at December 31 2013, whichever came first. Information on neoadjuvant and adjuvant treatment and recurrence was obtained from patient records. All haematoxylin & eosin stained slides from all cases were re-evaluated by one pathologist (JEL), blinded to the original report and outcome, as previously described, to get a uniform assessment of all histopathological parameters.

All EU and national regulations and requirements for handling human samples have been fully complied with during the conduct of this project; i.e. decision no. 1110/94/EC of the European Parliament and of the Council (OJL126 18,5,94), the Helsinki Declaration on ethical principles for medical research involving human subjects, and the EU Council Convention on human rights and Biomedicine. The study was approved of by the Ethics committee of Lund University (ref no 445/07), whereby the committee waived the need for consent other than by the option to opt out. All information from the patient records was anonymized and de-identified prior to analysis.

### Tissue microarray construction

Tissue microarrays (TMAs) were constructed using a semi-automated arraying device (TMArrayer, Pathology Devices, Westminister, MD, USA). A standard set of three tissue cores (1 mm) were obtained from each of the 175 formalin fixated paraffin embedded primary tumours and from lymph node metastases from 105 of the cases, whereby one to three lymph node metastases were sampled in each case.

### Immunohistochemistry, Silver In-Situ Hybridization and staining evaluation

All immunohistochemical staining and *in situ* hybridization (ISH) was performed on 4 μm TMA-sections. Immunohistochemistry for EGFR and HER3 was performed in an Autostainer Plus (Dako, Glostrup, Denmark) after automated pre-treatment with the PT-link system (Dako), using the monoclonal anti-EGFR antibody 31G7 (Zymed Laboratories Inc, San Francisco, CA, USA), diluted 1:25 and the monoclonal anti-HER3 antibody SP71 (Novus Biologicals LTD, Cambridge, UK), diluted 1:100, respectively. HER2 immunohistochemistry was performed on a BenchMark ULTRA instrument (Ventana Medical Systems, Inc. Tucson, AZ, USA). ULTRA Cell Conditioning (ULTRA CC1), pH9, was used for heat induced epitope retrieval (HIER). The monoclonal primary antibody PATHWAY anti-HER-2/neu (4B5), (Ventana Medical Systems, Inc.) was incubated for 20 minutes and the antibody-antigen complex was visualized with ultraView Universal DAB Detection kit (Ventana Medical Systems, Inc.)

HER2 ISH was also performed on the BenchMark ULTRA instrument (Ventana Medical Systems, Inc.). The peptide bonds were broken with ULTRA Cell Conditioning (ULTRA CC2), pH6, and ISH protease3. HER2 gene and Chromosome 17 (Chr17) were detected with INFORM HER2 Dual ISH DNA Probe Cocktail Assay (Ventana Medical Systems, Inc.). For visualization, ultraView SISH DNP Detection Kit and ultraView Red ISH DIG Detection Kit (Ventana Medical Systems, Inc.) were used, giving black and red chromogenic signals. As a final step, all slides were counterstained with haematoxylin.

The herein used anti-HER3 antibody has been validated by siRNA-mediated knockdown, immunocytochemistry and quantitative real-time PCR [35].

The immunohistochemical staining of EGFR, HER2 and HER3 was annotated by two independent observers (JEL and MH for EGFR, JEL and CFW for HER2/3) and consensus was reached in discordant cases.

EGFR, HER2 and HER3 protein expression was evaluated using the recommended protocol for HER2 testing in gastric and gastroesophageal junction cancer biopsies [42], taking complete, basolateral, or lateral membranous reactivity in a minimum of 5 clustered positive cancer cells into account, with the intensity recorded as 0, 1+, 2+ or 3+. Cytoplasmic staining was denoted as a separate category, but grouped with 1+ in the statistical analyses. Protein expression was grouped 0–2+ vs 3+, whereby 0–2+ was regarded as low expression and 3+ as high expression.

Assessment of ISH was performed according to the Ventana INFORM HER2 Dual ISH DNA Probe Cocktail Assay Interpretation Guide, and annotated by one pathologist (JEL). For each core 20 cancer cells were counted, and if the resulting HER2/Chr17 ratio fell within 1.5 and 2.5, another 20 cells were counted. A ratio above 2.0 was denoted as amplified. Assessment of HER2 ISH was only performed on cases that had either 2+ or 3+ immunohistochemical HER2 expression.

### Statistical analysis

Chi square test was applied to analyse the relationship between the dichotomized expression of each biomarker and clinicopathological parameters. Two patients with PB-type adenocarcinomas who had received neoadjuvant chemotherapy were excluded from the correlation and survival analyses. Three additional patients were excluded from the survival analyses; two with I-type adenocarcinomas who died within one month from surgery due to complications and one with PB-type adenocarcinoma who emigrated 5 months after surgery.

Kaplan Meier estimates of 5-year RFS and OS and log rank test were applied to evaluate survival differences in strata according to high and low expression of each biomarker. For PB-type tumours, biomarker expression was also combined with given adjuvant treatment; gemcitabine vs none/other. To estimate the interaction between given adjuvant treatment and biomarker expression in relation to survival, the following interaction variable was constructed; gemcitabine-based adjuvant treatment (+/−) × biomarker (high/low). Hazard ratios (HR) and 95% confidence intervals (CI) for death and recurrence within 5 years were calculated by Cox regression proportional hazard’s modelling in unadjusted analysis and in a multivariable model adjusted for differentiation grade, T-stage, N-stage, perineural invasion, lymphatic invasion, vascular invasion, invasion in peripancreatic fat and adjuvant chemotherapy. A backward conditional method was used for variable selection in the adjusted model. The proportional hazards assumption was tested by examining log-log survival curves.

All tests were two sided. P-values <0.05 were considered significant, and no adjustments were made for the number of tests performed. All statistical analyses were performed using IBM SPSS Statistics version 22.0 (SPSS Inc., Chicago, IL, USA).

## Results

### Distribution of protein expression of EGFR, HER2 and HER3 and *HER2* gene amplification

**Fig 1:**
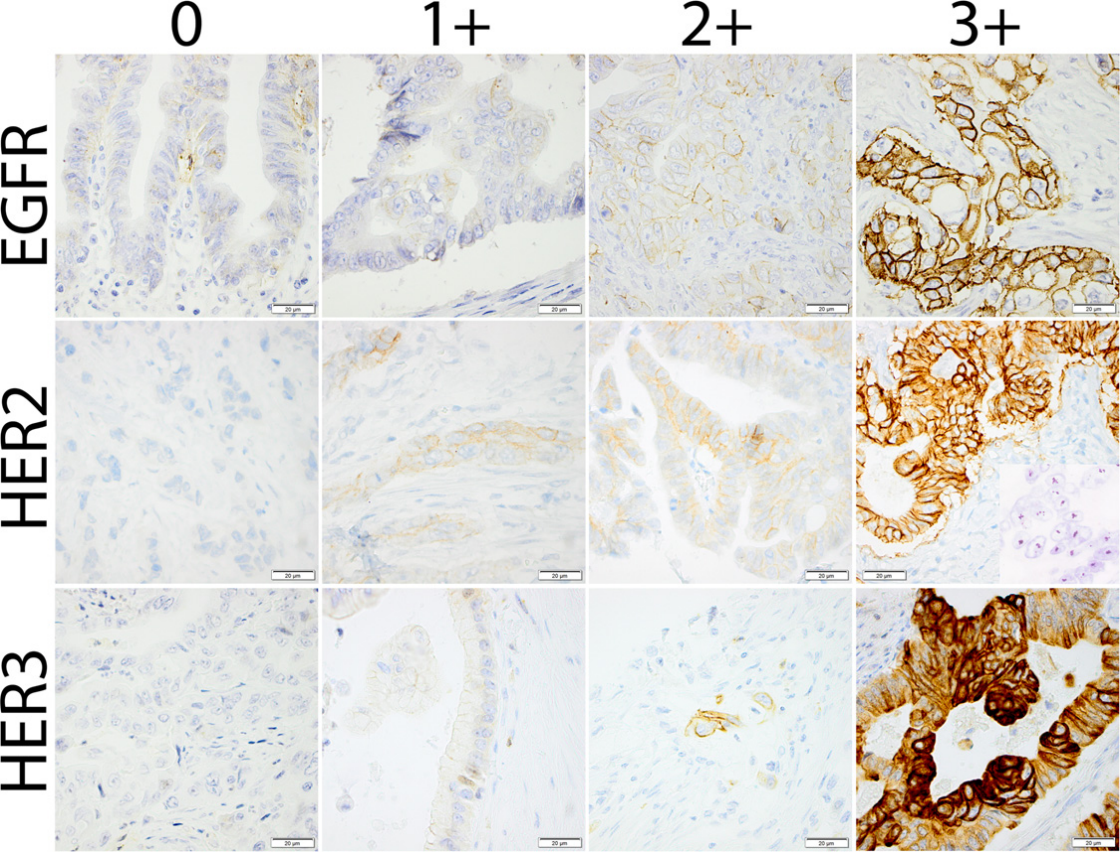
Sample immunohistochemical images. Photomicrographs representing different categories of immunohistochemical staining for EGFR, HER2 and HER3, respectively. An image visualizing *HER2* gene amplification by silver in situ hybridization is shown together with the HER2 3+ case.

Examples of immunohistochemistry scores 0, 1+, 2+ and 3+ for EGFR, HER2 and HER3 expression and *HER2* gene amplification by SISH are shown in Fig 1.

The distribution of expression of EGFR, HER2 and HER3 in PB- and I-type primary tumours is shown in Table 1. For cases with 2+ or 3+ expression of HER2, the results of SISH for *HER2* are also shown. The fraction of cases with 3+ expression differed significantly between PB-type and I-type tumours for HER3 (17% vs 51%, p<0.001), and for HER2 (0% vs 6%, p = 0.017), but not for EGFR. HER2 2+ expression was seen in 14% of both PB- and I-type tumours. SISH for *HER2* failed in 46% of the cases, probably due to prolonged tissue fixation in formaldehyde. All assessable HER2 3+ cases showed amplification of *HER2*, as did one 2+ PB-type case of ampullary origin. In total, 7% (5/68) of ampullary adenocarcinoma and 8% (4/49) of I-type ampullary adenocarcinoma showed HER2 overexpression (either immunohistochemistry 3+ or SISH+). There were no cases with 3+ co-expression of all three HER family members.

**Table 1:** Expression of EGFR, HER2 and HER3, and amplification status for *HER2* in pancreatobiliary and intestinal type periampullary adenocarcinoma.

		PB-type	I-type	All
		n = 110	n = 65	n = 175
Excluded due to neoadjuvant treatment		2	0	2
EGFR IHC score				
	0	2 (2%)	2 (3%)	4 (2%)
	1	18 (17%)	16 (25%)	34 (20%)
	2	39 (36%)	22 (35%)	61 (36%)
	3	48 (45%)	23 (37%)	71 (42%)
	Unassessable	1	2	3
HER2 IHC score				
	0	48 (44%)	27 (43%)	75 (44%)
	1	45 (42%)	23 (37%)	68 (40%)
	2	15 (14%)	9 (14%)	24 (14%)
	3	0 (0%)	4 (6%)	4 (2%)
	Unassessable	0	2	2
HER2 SISH (for IHC 2/3+)				
	Not amplified	8 (53%)	4 (31%)	12 (43%)
	Amplified	1 (7%)	2 (15%)	3 (11%)
	Failed SISH	6 (40%)	7 (54%)	13 (46%)
	Not assessed	93	52	145
HER3 IHC score				
	0	67 (63%)	18 (29%)	85 (50%)
	1	18 (17%)	12 (19%)	30 (18%)
	2	4 (4%)	1 (2%)	5 (3%)
	3	18 (17%)	32 (51%)	50 (29%)
	Unassessable	1	2	3

IHC: immunohistochemistry. SISH: silver in situ hybridization. Due to rounding effects, percentages may not add up to 100.

Since there were no tumours with 3+ expression of HER2 in the PB-group, further analyses on associations and prognosis related to 3+ expression of HER2 in PB-type tumours could not be done.

In the full cohort there were 86 cases with evaluable stainings from both primary tumour and corresponding lymph node metastases. There were no significant differences in the proportion of 3+ expression of EGFR, HER2 or HER3 between primary tumours and corresponding metastases (data not shown).

### Associations between EGFR, HER2 and HER3 protein expression and clinicopathological parameters

In PB-type adenocarcinomas there were no significant associations between 3+ expression of EGFR or HER3 and clinicopathological parameters (S1 Table).

In I-type adenocarcinomas, high EGFR expression was significantly associated with larger tumour size, but not with any other parameter. HER2 expression was not associated with any parameter. For HER3, there was an inverse association between high protein expression and tumour stage, perineural growth, blood vessel invasion, growth in peripancreatic fat and recurrence (S2 Table). When considering I-type adenocarcinomas of ampullary origin only, thus excluding duodenal origin, the associations remained significant for tumour stage (p<0.001), perineural growth (p = 0.002) and growth in peripancreatic fat (p<0.001) (data not shown).

### Impact of EGFR, HER2 and HER3 expression on 5-year recurrence-free and overall survival

**Fig 2:**
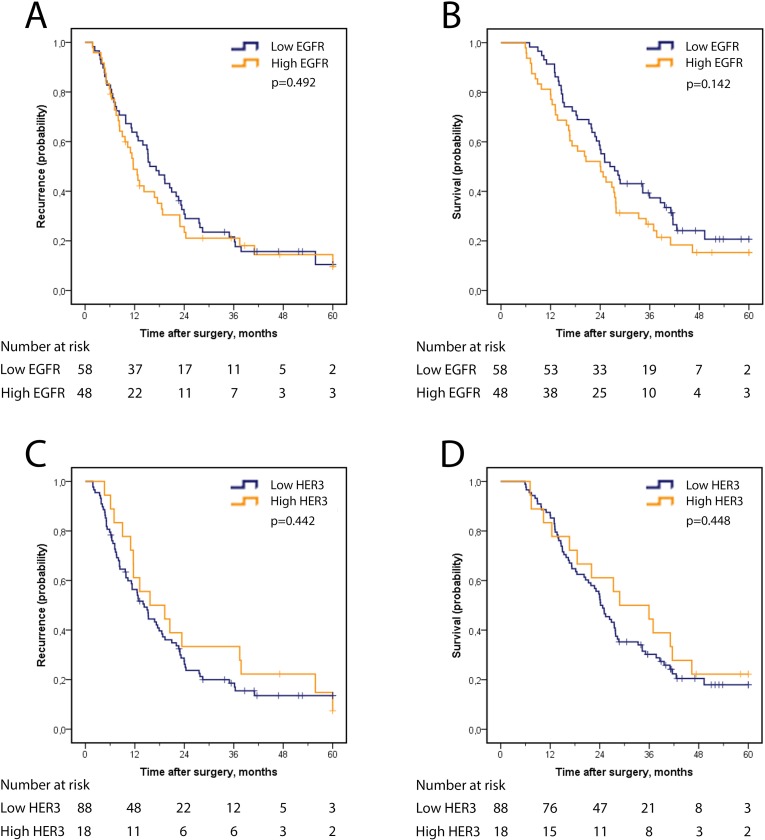
Recurrence-free and overall survival according to EGFR and HER3 expression in PB-type adenocarcinoma. Kaplan Meier analysis of five-year recurrence-free survival in strata according to high and low expression of (A) EGFR and (C) HER3 and overall survival according to high and low expression of (B) EGFR and (D) HER3.

**Fig 3:**
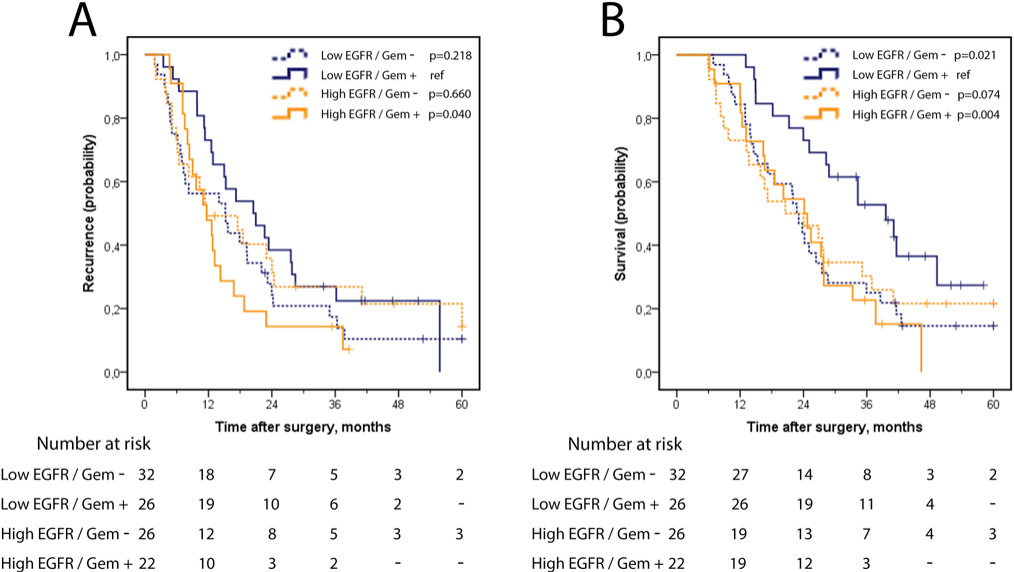
Recurrence-free and overall survival in strata according to EGFR expression and adjuvant gemcitabine in PB-type adenocarcinoma. Kaplan Meier analysis of (A) five-year recurrence-free survival and (B) overall survival in combined strata according to EGFR expression (high/low) and adjuvant gemcitabine (yes/no).

In the full group of PB-type cases, recurrence-free survival (RFS) and OS did not differ by expression of EGFR or HER3 (Fig 2A–2D). Analysis in strata according to adjuvant treatment, however, revealed a significantly reduced RFS and OS for patients that had received adjuvant gemcitabine and had tumours with high, as compared with low, EGFR expression (Fig 3A and 3B), whereas no survival difference was seen according to high or low EGFR expression among untreated PB-type cases (Fig 3). As further shown in Table 2, there was a significant treatment interaction between EGFR expression and adjuvant gemcitabine in relation to OS (p_interaction_ = 0.042), but not in relation to RFS. When considering only pancreatic tumour origin, a significantly shorter RFS (HR 2.47, 95% CI 1.02–6.00) and OS (HR 3.47, 95% CI 1.34–8.97) was seen in adjuvant gemcitabine treated cases with tumours displaying high EGFR expression, as compared with low EGFR expression, whereas no survival difference was seen according to high or low EGFR expression among cases not receiving adjuvant gemcitabine. There was however no significant interaction in relation to neither RFS, p(_interaction_) = 0.084 nor OS, p(_interaction_) = 0.160.

**Table 2:** Cox proportional hazards analysis of the impact of EGFR expression on recurrence-free and overall survival in strata according to adjuvant gemcitabine in patients with PB-type adenocarcinoma.

		RFS		OS	
		HR (95% CI)	p(interaction)	HR (95% CI)	p(interaction)
Gem -					
Low EGFR (n = 32)		1.00		1.00	
High EGFR (n = 26)		0.84 (0.47–1.49)	0.098	0.94 (0.53–1.68)	**0.042**
Gem +					
Low EGFR (n = 26)		1.00		1.00	
High EGFR (n = 22)		**1.93 (1.02–3.65)**		**2.69 (1.34–5.42)**	

Bold text indicates significant values

**Fig 4:**
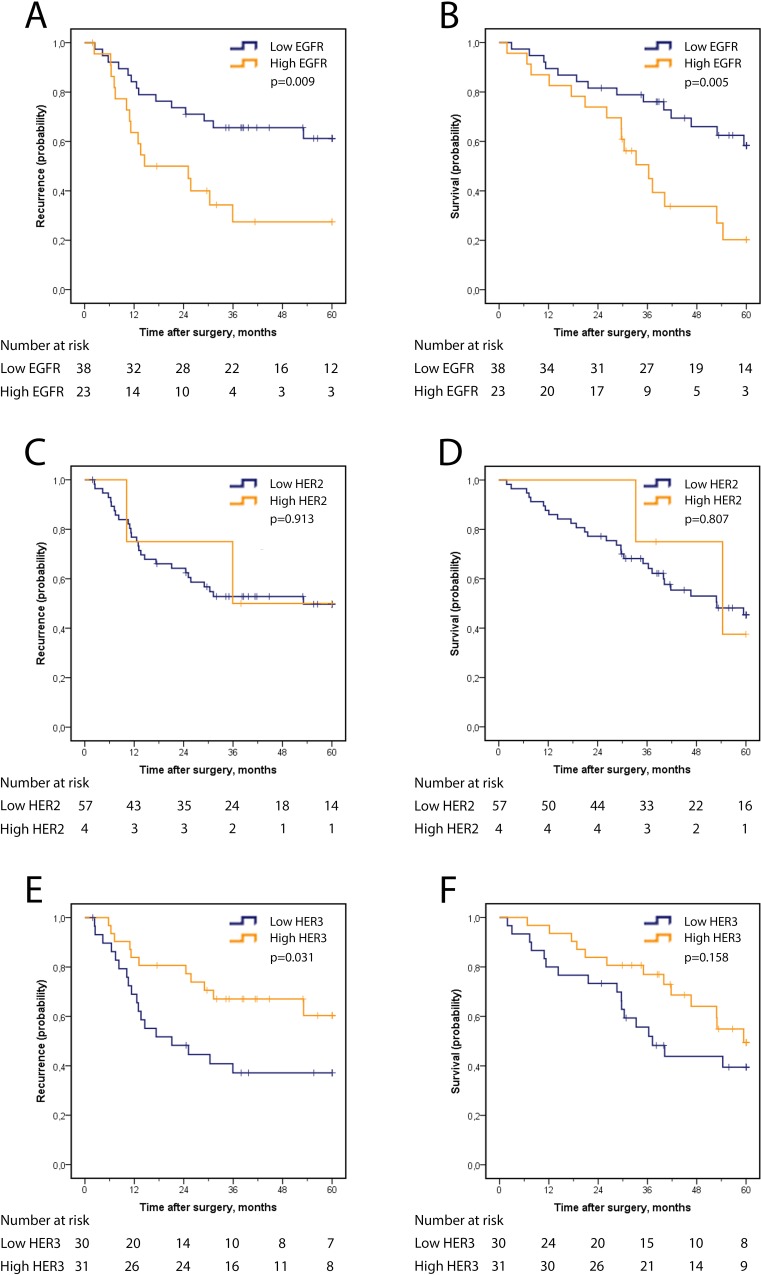
Recurrence-free and overall survival according to EGFR, HER2 and HER3 expression in I-type adenocarcinoma. Kaplan Meier analysis of five-year recurrence-free survival in strata according to high and low expression of (A) EGFR, (C) HER2, (E) HER3 and overall survival according to high and low expression of (B) EGFR, (D) HER2, and (F) HER3.

In I-type cases, Kaplan Meier analysis revealed a significantly shorter OS and RFS for cases with high EGFR expression (Fig 4A and 4B). Significance was retained in univariable Cox regression analysis for RFS (HR 2.58, 95% CI 1.23–5.38) and OS (HR 2.74, 95% CI 1.32–5.69), but not in multivariable analysis (Table 3). The prognostic value of EGFR expression did not differ according to adjuvant treatment in I-type tumours (data not shown), and there was no significant difference in RFS or OS between cases with high or low HER2 expression (Fig 4C and 4D and Table 3). There was a significant association between high HER3 expression and a longer RFS (p = 0.031), and significance was retained in univariable Cox regression analysis (HR 0.45, 95% CI 0.21–0.95), but not in multivariable analysis (Table 3). There was no significant association between HER3 expression and OS.

**Table 3:** Cox proportional hazards analysis of the impact of EGFR, HER2 and HER3 expression on recurrence-free and overall survival in patients with I-type adenocarcinoma.

	RFS			OS		
	n (events)	Univariable	Multivariable	n (events)	Univariable	Multivariable
EGFR						
Low	38 (14)	1.00	1.00	38 (14)	1.00	1.00
High	23 (15)	**2.58 (1.23–5.38)**	1.80 (0.85–3.82)	23 (16)	**2.74 (1.32–5.69)**	1.55 (0.68–3.53)
HER2						
Low	57 (27)	1.00		57 (28)	1.00	
High	4 (2)	0.92 (0.22–3.89)	NI	4 (2)	0.84 (0.20–3.51)	NI
HER3						
Low	30 (18)	1.00	1.00	30 (17)	1.00	
High	31 (11)	**0.45 (0.21–0.95)**	1.37 (0.52–3.62)	31 (13)	0.60 (0.29–1.23)	NI

Multivariable analysis adjusted for tumour grade, N-stage (N0 vs N1), T-stage (T1-2 vs T3-4), perineural growth, lymphatic invasion, blood vessel invasion, invasion of perineural fat and adjuvant treatment. NI, not investigated. Bold text indicates significant values.

## Discussion

In this study, we have evaluated the prognostic impact of EGFR, HER2 and HER3 in periampullary adenocarcinoma, by morphological type and adjuvant treatment. In intestinal-type adenocarcinoma, we found that high HER3 expression was a favourable prognostic factor and that high EGFR expression was an adverse prognostic factor, although none of these associations were independent of other prognostic factors. In addition, in pancreatobiliary-type tumours, EGFR expression was found to be an adverse prognostic factor only in cases that received adjuvant gemcitabine, and a positive effect of gemcitabine was only seen in cases with low EGFR expression, with a significant interaction between EGFR expression and adjuvant gemcitabine in relation to overall survival. HER2 expression was not prognostic, neither in intestinal-type, nor in pancreatobiliary-type tumours.

Our results regarding the expression of EGFR, HER2 and HER3 are comparable to previously published results, with the possible exception of EGFR expression in I-type adenocarcinomas, where we found 3+ expression in 38% of the cases, compared with 4% expression [14] and 19% 3+ expression [15] in other studies. In addition, we were not able to demonstrate the previously described association between HER2 and HER3 expression. Our results regarding the adverse prognostic effect of EGFR in the entire group of intestinal-type, but not in pancreatobiliary type, adenocarcinoma confirm the results of Xia et al [15], and further underscore the biological differences between pancreatobiliary and intestinal type periampullary adenocarcinomas. Our finding of a positive effect of gemcitabine only in cases with low EGFR expression has to our knowledge not been shown in these tumour types before. This finding is however compatible with the described increase in EGFR expression in colon cancer cell lines when resistance to chemotherapy was induced [43], and a better response to chemoradiotherapy in esophageal squamous cell carcinoma displaying low EGFR expression [44]. Our finding thus suggests that gemcitabine has a limited or no effect on survival in pancreatobiliary type tumours with high expression of EGFR. From a mechanistic viewpoint, inhibition of EGFR could theoretically seem like an attractive treatment option in these patients. However, immunohistochemical assessment of EGFR expression has not been a good predictor of response to the EGFR antibodies cetuximab or panitumumab in colorectal cancer, compared with *EGFR* copy number and *KRAS* mutation analysis [45–48]. Expression of EGFR also failed to predict response to the EGFR tyrosine kinase inhibitor erlotinib, when added to gemcitabine in the NCIC CTG PA.3 trial, in patients with locally advanced or metastatic pancreatic cancer [13]. Although the herein studied cohort contains both adjuvant treated and untreated patients, thus enabling identification of potential predictive biomarkers, firm conclusions on treatment prediction should not be drawn, given the retrospective character of the cohort. Another caveat is that several tests have been made in the present study, which increases the risk for type I errors, i.e. detecting a difference that is coincidental.

Another possible limitation to the present study is the use of tissue microarrays, whereby the issue of representativity in relation to whole tissue sections may always be raised. One should however bear in mind that whole tissue sections also represent only a minor part of the tumour, and that the tissue microarray technique allows sampling from different regions in different tissue blocks, thus enabling detection of heterogeneous expression. With a few exceptions [49], the tissue microarray method accurately reflects the expression of different proteins, and is a well-validated platform for studies of biomarkers [50].

Our results on the incidence of HER2 overexpression (3+ and/or ISH amplification) are well in line with previous results, with a low frequency in pancreatobiliary-type tumours or pancreatic cancer, and a higher frequency in I-type adenocarcinomas, although the latter is lower than in gastric cancer [42]. The HER2-HER3 homodimer is a powerful activator of the PI3K/Akt pathway [51], causing aberrant proliferative and antiapoptotic intracellular signals [52]. Inhibition of HER2 activity, however, causes upregulation of HER3, and simultaneous blockage of HER2 and HER3 activity gives a more potent inhibition of HER2 dependent oncogenic features than blockage of one receptor alone [53]. In line with those findings there is one case report of second line therapy with the antibodies trastuzumab and pertuzumab, thus inhibiting both HER2 and its dimerization, for HER2 overexpressing metastatic ampullary cancer, describing stable disease, with some shrinkage of metastases, and a longer survival than expected upon standard chemotherapy [54]. Blockage of HER3 activity is thus of interest, and clinical trials with the HER3 antibody patritumab are ongoing [55, 56].

Our finding of a longer recurrence-free survival in I-type cases with high HER3 expression is unexpected, given the oncogenic features of HER3, and in contrast with previous reports on various non-gastrointestinal cancers [31], one on colon cancer [57], and two on gastric cancer [58, 59], but harmonize with a few studies on breast [34], colorectal [32, 33], and gastric and oesophageal cancer [35]. A possible explanation for our finding could be that high HER3 expression reflects a less proliferative tumour, which is in line with the described expression of HER3 in non-proliferating parts of colon epithelium and colon cancer [60]. Another explanation for the diverging results regarding the prognostic effect of HER3 could also be the use of different antibodies, and algorithms for assessing the expression. The antibody used in the present study is however well validated [35], and we have used the well-known protocol for assessing HER2-immunohistochemistry in biopsies of gastric cancer, to make the annotation easily reproducible.

In the current study, the least studied HER family member, HER4, was not included, but given the complex network of signalling pathways that combinations of HER dimers and ligands can activate, it is not unlikely that expression of HER4 may have prognostic or predictive implications in periampullary adenocarcinoma.

In summary, the results from the present study demonstrate that high EGFR expression is an unfavourable prognostic factor in in gemcitabine treated pancreatobiliary type adenocarcinoma. The finding of a potential interaction between EGFR expression and response to adjuvant gemcitabine in pancreatobiliary type tumours is novel and of potential clinical relevance, and therefore merits confirmation and further study, both in a mechanistic context as well as in additional patient cohorts. EGFR expression was also an unfavourable prognostic factor, although not independent from other factors, in intestinal type tumours. Expression of HER3 was found to differ between pancreatobiliary and intestinal type adenocarcinomas and to be a favourable prognostic factor, however not independent, in intestinal type adenocarcinoma. Overexpression of HER2 was observed in 8% of intestinal type ampullary adenocarcinoma, and was not associated with prognosis. It is feasible that further steps towards individualized therapy in periampullary adenocarcinoma will involve simultaneous targeting of several members of the HER family.

## Supporting Information

S1 Table Associations between expression of EGFR, HER3 and clinicopathological parameters in pancreatobiliary-type periampullary adenocarcinoma. M, median. IQR, interquartile range.
(DOCX)

S2 Table Associations between expression of EGFR, HER2, HER3 and clinicopathological parameters in intestinal-type periampullary adenocarcinoma. M, median. IQR, interquartile range. Bold text indicates significant values.
(DOCX)
