# Supplementary material for: Expression and Prognostic Significance of Human Epidermal Growth Factor Receptors 1, 2 and 3 in Periampullary Adenocarcinoma
Source: PLoS One. 2016 Apr 12;11(4):e0153533. doi: 10.1371/journal.pone.0153533 (PMC4829175; doi:10.1371/journal.pone.0153533)
Supplement: S1 Table — M, median. IQR, interquartile range. (DOCX) [file pone.0153533.s001.docx]

|  |  | EGFR | | | HER3 | | |
| --- | --- | --- | --- | --- | --- | --- | --- |
|  |  | low, 0-2+  (n=60) | high, 3+  (n=49) | p-value | low, 0-2+  (n=90) | high, 3+  (n=19) | p-value |
| Excluded, neoadjuvant treatment | | 1 | 1 |  | 1 | 1 |  |
| Lost to follow up | | 1 |  |  | 1 |  |  |
| EGFR | |  |  |  |  |  | 0.194 |
|  | low |  |  |  | 51 (88%) | 7 (12%) |  |
|  | high |  |  |  | 37 (77%) | 11 (23%) |  |
| HER3 | |  |  | 0.194 |  |  |  |
|  | low | 51 (58%) | 37 (42%) |  |  |  |  |
|  | high | 7 (39%) | 11 (61%) |  |  |  |  |
| Year of surgery, M (IQR) | | 2009 (2005-2010) | 2009 (2005-2010) | 0.206 | 2009 (2006-2010) | 2007 (2005-2010) | 0.329 |
| Age, M (IQR) | | 67 (62-73) | 67 (61-73) | 0.615 | 66 (61-73) | 70 (65-74) | 0.402 |
| Sex | |  |  | 0.118 |  |  | 1.000 |
|  | Women | 23 (46%) | 27 (54%) |  | 42 (84%) | 8 (16%) |  |
|  | Men | 35 (62%) | 21 (38%) |  | 46 (82%) | 10 (18%) |  |
| Tumour origin | |  |  | 0.058 |  |  | 0.055 |
|  | Ampulla Vateri | 15 (79%) | 4 (21%) |  | 19 (100%) | 0 (0%) |  |
|  | Distal bile duct | 21 (48%) | 23 (52%) |  | 36 (82%) | 8 (18%) |  |
|  | Pancreas | 22 (51%) | 21 (49%) |  | 33 (77%) | 10 (23%) |  |
| Tumour size, mm, M (IQR) | | 30 (25-35) | 30 (23-35) | 0.992 | 30 (25-35) | 30 (21-40) | 0.068 |
| Differentiation grade | |  |  | 0.071 |  |  | 0.593 |
|  | Well / moderate | 26 (67%) | 13 (33%) |  | 31 (79%) | 8 (21%) |  |
|  | Poor | 32 (48%) | 35 (52%) |  | 57 (85%) | 10 (15%) |  |
| T-stage | |  |  | 0.218 |  |  | 0.119 |
|  | T1 / T2 | 9 (75%) | 3 (25%) |  | 8 (67%) | 4 (33%) |  |
|  | T3 / T4 | 49 (52%) | 45 (48%) |  | 80 (85%) | 14 (15%) |  |
| N-stage | |  |  | 0.827 |  |  | 0.087 |
|  | N0 | 15 (52%) | 14 (48%) |  | 21 (72%) | 8 (28%) |  |
|  | N1 | 43 (56%) | 34 (44%) |  | 67 (87%) | 10 (13%) |  |
| Perineural growth | |  |  | 0.638 |  |  | 0.054 |
|  | No | 11 (50%) | 11 (50%) |  | 15 (68%) | 7 (32%) |  |
|  | Yes | 47 (56%) | 37 (44%) |  | 73 (87%) | 11 (13%) |  |
| Growth in lymphatic vessels | |  |  | 0.835 |  |  | 0.782 |
|  | No | 17 (53%) | 15 (47%) |  | 26 (81%) | 6 (19%) |  |
|  | Yes | 41 (55%) | 33 (45%) |  | 62 (84%) | 12 (16%) |  |
| Growth in blood vessels | |  |  | 0.306 |  |  | 0.413 |
|  | No | 41 (59%) | 29 (41%) |  | 60 (86%) | 10 (14%) |  |
|  | Yes | 17 (47%) | 19 (53%) |  | 28 (78%) | 8 (22%) |  |
| Growth in peripancreatic fat | |  |  | 0.810 |  |  | 0.523 |
|  | No | 13 (59%) | 9 (41%) |  | 17 (77%) | 5 (23%) |  |
|  | Yes | 45 (54%) | 39 (46%) |  | 71 (85%) | 13 (15%) |  |
| Margins | |  |  | 0.687 |  |  | 0.269 |
|  | R0 | 4 (67%) | 2 (33%) |  | 4 (67%) | 2 (33%) |  |
|  | R1/Rx | 54 (54%) | 46 (46%) |  | 84 (84%) | 16 (16%) |  |
| Adjuvant treatment | |  |  | 1.000 |  |  | 0.796 |
|  | No gemcitabine | 32 (55%) | 26 (45%) |  | 49 (84%) | 9 (16%) |  |
|  | Gemcitabine | 26 (54%) | 22 (46%) |  | 39 (81%) | 9 (19%) |  |
| Recurrence | |  |  | 0.929 |  |  | 0.636 |
|  | None | 9 (50%) | 9 (50%) |  | 16 (89%) | 2 (11%) |  |
|  | Local | 16 (55%) | 13 (45%) |  | 25 (86%) | 4 (14%) |  |
|  | Distant | 33 (56%) | 26 (44%) |  | 47 (80%) | 12 (20%) |  |
