# Supplementary material for: Expression and Prognostic Significance of Human Epidermal Growth Factor Receptors 1, 2 and 3 in Periampullary Adenocarcinoma
Source: PLoS One. 2016 Apr 12;11(4):e0153533. doi: 10.1371/journal.pone.0153533 (PMC4829175; doi:10.1371/journal.pone.0153533)
Supplement: S2 Table — M, median. IQR, interquartile range. Bold text indicates significant values. (DOCX) [file pone.0153533.s002.docx]

|  |  | EGFR | | | HER2 | | | HER3 | | |
| --- | --- | --- | --- | --- | --- | --- | --- | --- | --- | --- |
|  |  | low, 0-2+ (n=40) | high, 3+ (n=23) | p-value | low, 0-2+ (n=59) | high, 3+ (n=4) | p-value | low, 0-2+ (n=31) | high, 3+ (n=32) | p-value |
| Excluded, death within 1 month | | 2 | 0 |  | 2 | 0 |  | 1 | 1 |  |
| Lost to follow up | | 0 | 0 |  | 0 | 0 |  | 0 | 0 |  |
| EGFR | |  |  |  |  |  | 0.628 |  |  | 0.192 |
|  | low |  |  |  | 36 (95%) | 2 (5%) |  | 16 (42%) | 22 (58%) |  |
|  | high |  |  |  | 21 (91%) | 2 (9%) |  | 14 (61%) | 9 (39%) |  |
| HER2 | |  |  | 0.628 |  |  |  |  |  | 0.354 |
|  | low | 36 (63%) | 21 (37%) |  |  |  |  | 27 (47%) | 30 (53%) |  |
|  | high | 2 (50%) | 2 (50%) |  |  |  |  | 3 (75%) | 1 (25%) |  |
| HER3 | |  |  | 0.192 |  |  | 0.354 |  |  |  |
|  | low | 16 (53%) | 14 (47%) |  | 27 (90%) | 3 (10%) |  |  |  |  |
|  | high | 22 (71%) | 9 (29%) |  | 30 (97%) | 1 (3%) |  |  |  |  |
| Year of surgery, M (IQR) | | 2006 (2003-2009) | 2008 (2005-2010) | 0.562 | 2007 (2004-2010) | 2007 (2002-2010) | 0.292 | 2006 (2003-2009) | 2007 (2005-2010) | 0.670 |
| Age, M (IQR) | | 67 (59-71) | 66 (62-69) | 0.754 | 66 (60-70) | 56 (46-74) | 0.295 | 65 (58-69) | 68 (60-71) | 0.885 |
| Sex | |  |  | 1.000 |  |  | 1.000 |  |  | 0.799 |
|  | Women | 21 (62%) | 13 (38%) |  | 32 (94%) | 2 (6%) |  | 16 (47%) | 18 (53%) |  |
|  | Men | 17 (63%) | 10 (37%) |  | 25 (93%) | 2 (7%) |  | 14 (52%) | 13 (48%) |  |
| Tumour origin | |  |  | 1.000 |  |  | 0.569 |  |  | 0.534 |
|  | Duodenum | 8 (62%) | 5 (38%) |  | 13 (100%) | 0 (0%) |  | 5 (38%) | 8 (62%) |  |
|  | Ampulla, intestinal type | 30 (62%) | 18 (38%) |  | 44 (92%) | 4 (8%) |  | 25 (52%) | 23 (48%) |  |
| Tumour size, mm, M (IQR) | | 25 (15-40) | 30 (15-40) | **0.020** | 30 (15-40) | 23 (16-29) | 0.946 | 30 (23-40) | 20 (13-40) | 0.118 |
| Differentiation grade | |  |  | 0.114 |  |  | 0.612 |  |  | 0.204 |
|  | Well / moderate | 22 (73%) | 8 (27%) |  | 29 (97%) | 1 (3%) |  | 12 (40%) | 18 (60%) |  |
|  | Poor | 16 (52%) | 15 (48%) |  | 28 (90%) | 3 (10%) |  | 18 (58%) | 13 (42%) |  |
| T-stage | |  |  | 0.214 |  |  | 0.565 |  |  | **0.005** |
|  | T1 / T2 | 11 (79%) | 3 (21%) |  | 14 (100%) | 0 (0%) |  | 2 (14%) | 12 (86%) |  |
|  | T3 / T4 | 27 (57%) | 20 (43%) |  | 43 (91%) | 4 (9%) |  | 28 (60%) | 19 (40%) |  |
| N-stage | |  |  | 0.440 |  |  | 0.618 |  |  | 0.309 |
|  | N0 | 19 (58%) | 14 (42%) |  | 30 (91%) | 3 (9%) |  | 14 (42%) | 19 (58%) |  |
|  | N1/N2 | 19 (68%) | 9 (32%) |  | 27 (96%) | 1 (4%) |  | 16 (57%) | 12 (43%) |  |
| Perineural growth | |  |  | 0.394 |  |  | 0.582 |  |  | **0.013** |
|  | No | 28 (67%) | 14 (33%) |  | 40 (95%) | 2 (5%) |  | 16 (38%) | 26 (62%) |  |
|  | Yes | 10 (53%) | 9 (47%) |  | 17 (89%) | 2 (11) |  | 14 (74%) | 5 (26%) |  |
| Growth in lymphatic vessels | |  |  | 0.440 |  |  | 0.618 |  |  | 0.444 |
|  | No | 19 (68%) | 9 (32%) |  | 27 (96%) | 1 (4%) |  | 12 (43%) | 16 (57%) |  |
|  | Yes | 19 (58%) | 14 (42%) |  | 30 (91%) | 3 (9%) |  | 18 (55%) | 15 (45%) |  |
| Growth in blood vessels | |  |  | 1.000 |  |  | 1.000 |  |  | **0.024** |
|  | No | 35 (62%) | 21 (38%) |  | 52 (93%) | 4 (7%) |  | 25 (45%) | 31 (55%) |  |
|  | Yes | 3 (60%) | 2 (40%) |  | 5 (100%) | 0 (0%) |  | 5 (100%) | 0 (0%) |  |
| Growth in peripancreatic fat | |  |  | 0.103 |  |  | 0.602 |  |  | **<0.001** |
|  | No | 28 (70%) | 12 (30%) |  | 38 (95%) | 2 (5%) |  | 13 (33%) | 27 (67%) |  |
|  | Yes | 10 (48%) | 11 (52%) |  | 19 (90%) | 2 (10%) |  | 17 (81%) | 4 (19%) |  |
| Margins | |  |  | 0.075 |  |  | 1.000 |  |  | 1.000 |
|  | R0 | 14 (82%) | 3 (18%) |  | 16 (94%) | 1 (6%) |  | 8 (47%) | 9 (53%) |  |
|  | R1/Rx | 24 (55%) | 20 (45%) |  | 41 (93%) | 3 (7%) |  | 22 (50%) | 22 (50%) |  |
| Adjuvant treatment | |  |  | 0.775 |  |  | 1.000 |  |  | 0.270 |
|  | No adjuvant | 26 (60%) | 17 (40%) |  | 40 (93%) | 3 (7%) |  | 19 (44%) | 24 (56%) |  |
|  | Any adjuvant | 12 (67%) | 6 (33%) |  | 17 (94%) | 1 (6%) |  | 11 (61%) | 7 (39%) |  |
| Recurrence | |  |  | 0.073 |  |  | 1.000 |  |  | **0.036** |
|  | None | 24 (75%) | 8 (25%) |  | 30 (94%) | 2 (6%) |  | 12 (38%) | 20 (62%) |  |
|  | Local | 2 (50%) | 2 (50%) |  | 4 (100%) | 0 (0%) |  | 1 (25%) | 3 (75%) |  |
|  | Distant | 12 (48%) | 13 (52%) |  | 23 (92%) | 2 (8%) |  | 17 (68%) | 8 (32%) |  |
